# The Inflammatory Biomarkers Behavior Profile of Patients Following Elective Degenerative Spine Surgery and Differences Compared to Those Coursing With a Postoperative Spinal Infection: Protocol for a Systematic Review

**DOI:** 10.2196/41555

**Published:** 2023-04-28

**Authors:** José Alberto Israel Romero Rangel, Fiacro Jiménez Ponce, Rosa Maria Wong Chew, Juan Osvaldo Talavera Piña, Adolfo Martínez Tovar

**Affiliations:** 1 Spine Clinic Neurological Center The American-British Cowdray Medical Center IAP Mexico City Mexico; 2 National Autonomous University of Mexico Mexico City Mexico; 3 General Hospital of Mexico Mexico City Mexico; 4 Infectious Diseases Department National Autonomous University of Mexico Mexico City Mexico; 5 Reseach and Education Department The American-British Cowdray Medical Center IAP Mexico City Mexico; 6 Laboratory of Special Tests Hematology Department General Hospital of Mexico Mexico City Mexico

**Keywords:** biomarker behavior profile, biomarkers, C-reactive protein, degenerative spinal diseases, Dickkopf-1, erythrocyte sedimentation rate, inflammatory biomarker, inflammatory disease, neuroscience, postoperative spinal infection, procalcitonin, serum biomarkers, spinal infection, spinal surgical site infection, spine surgery, review, systematic review

## Abstract

**Background:**

The incidence of postoperative spinal infection (PSI) ranges from 0% to 10%, with devastating effects on the patient prognosis because of higher morbidity while increasing costs to the health care system. PSIs are elusive and difficult to diagnose, especially in the early postoperative state, because of confusing clinical symptoms, rise in serum biomarkers, or imaging studies. Current research on diagnosis has focused on serum biomarkers; nevertheless, most series rely on retrospective cohorts where biomarkers are studied individually and at different time points.

**Objective:**

This paper presents the protocol for a systematic review that aims to determine the inflammatory biomarker behavior profile of patients following elective degenerative spine surgery and their differences compared to those coursing with PSIs.

**Methods:**

The proposed systematic review will follow the PRISMA (Preferred Reporting Items for Systematic Reviews and Meta-Analyses) statement. This protocol was registered at PROSPERO on January 19, 2022. We will include studies related to biomarkers in adult patients operated on for degenerative spinal diseases and those developing PSIs. The following information will be extracted from the papers: (1) study title; (2) study author; (3) year; (4) evidence level; (5) research type; (6) diagnosis group (elective postoperative degenerative disease or PSI); (7a) region (cervical, thoracic, lumbosacral, and coccygeal); (7b) type of infection by anatomical or radiological site; (8) surgery type (including instrumentation or not); (9) number of cases; (10) mean age or individual age; (11) individual serum biomarker values from the preoperative state up to 90 days postoperative for both groups, including (10a) interleukin-6, (10b) presepsin, (10c) erythrocyte sedimentation rate, (10d) leukocyte count, (10e) neutrophil count, (10f) C-reactive protein, (10g) serum amyloid, (10h) white cell count, (10i) albumin, (10j) prealbumin, (10k) procalcitonin, (10l) retinol-associated protein, and (10m) Dickkopf-1; (11) postoperative days at symptoms or diagnosis; (12) type of organism; (13) day of starting antibiotics; (14) duration of treatment; and (15) any biases (including comorbidities, especially those affecting immunological status). All data on biomarkers will be presented graphically over time.

**Results:**

No ethical approval will be required, as this review is based on published data and does not involve interaction with human participants. The search for this systematic review commenced in February 2021, and we expect to publish the findings in mid-2023.

**Conclusions:**

This study will provide the behavior profile of biomarkers for PSI and patients following elective surgery for degenerative spinal diseases from the preoperative period up to 90 days postoperative, providing cutoff values on the day of diagnosis. This research will provide clinicians with highly trustable cutoff reference values for PSI diagnosis. Finally, we expect to provide a basis for future research on biomarkers that help diagnose more accurately and in a timely manner in the early stages of illness, ultimately impacting the patient’s physical and mental health, and reducing the disease burden.

**Trial Registration:**

PROSPERO CRD42022304645; https://www.crd.york.ac.uk/prospero/display_record.php?RecordID=304645

**International Registered Report Identifier (IRRID):**

DERR1-10.2196/41555

## Introduction

### Overview

The incidence of postoperative spinal infection (PSI) ranges from 0% to 10% depending on several factors related to the patient, the surgical intervention [1], and conditions of the health care system. PSI, otherwise called surgical site infection, has devastating effects on the patient prognosis because of higher morbidity [2] and mortality while increasing costs to the health care system [3]. PSI accounts for half of the readmissions following spinal surgery [3] and increases the cost of health care from US $150,000-$200,000 [4,5] per patient. Spinal infections affect the patient’s physical and mental health but also the health care system and the society by increasing the burden of economic cost and even indirect expenditures such as absence from work that will affect the patient’s capability to afford its treatment, making this a negative circle of factors that will ultimately affect the patient’s quality of life [6].

PSI occurs either by direct inoculation during surgery, by the incursion of skin pathogens through the skin incision [7], or because of hematogenous spread [8] where *Staphylococcus aureus* and *Escherichia coli* are the pathogens most frequently involved. Multiple studies have determined the clinical factors involved in the genesis of PSI, including diabetes, coronary artery disease, traumatic injuries, male gender, age >60 years, BMI>35, smoking, malnutrition, a score >3 in the American Society Anesthesiologist, a posterior approach, spinal fusion, prolonged surgical time, multilevel surgery, and transfusion [9-15]. Unfortunately, most patients requiring spinal surgery have these conditions, where multiple comorbidities increase the burden [16]. Therefore, preoperative evaluation and management of any preventable risk factor are of utmost importance to prevent PSI, which can lead to significant morbidity, sepsis, and even death [17].

PSIs require a high level of suspicion as they are difficult to diagnose [18], especially in the early postoperative state, because of many confounding factors. The mainstay for diagnosing de novo spine infection is a triad of clinical symptoms, serum tests (biomarkers and blood cultures), and correlative imaging [19]. All these 3 parameters are affected in PSI, as clinical symptoms (usually pain) can be attributed to postoperative pain and muscle contracture, rise in serum biomarkers can be explained by inflammatory changes or conditions associated with surgery, and early postoperative imaging studies such as simple or enhanced MRI cannot distinguish between these entities clearly as postoperative changes are not so different [18-20]. These situations lead us to rely on blood cultures [20] that often take 5 days to offer accurate results with high false negative rates, ultimately requiring reintervention with tissue sample as the last step in the diagnosis, where under challenging cases it is not infrequent to repeat the diagnostic protocol because of negative results on biopsy or failed response to medical treatment.

Current research has focused on serum biomarkers to detect spinal infection at the early stages of disease [21,22]; nevertheless, most rely on retrospective cohorts or case series and a scarcity of prospective cohorts. Adding to this problem is that serum biomarkers are studied individually and at different time points while having a heterogenous group of patients who had undergone spinal surgery because of a diversity of spine affections treated with different surgical procedures. Therefore, this protocol aims to collect all the available information currently investigated related to biomarkers and PSI and elective surgery for degenerative spinal disease in order to provide the biomarkers’ behavior profile from the preoperative day up to postoperative day 90 to guide differential diagnosis based on cutoff values among these groups.

We made a simple revision on PubMed before proceeding to the protocol and systematic review to look in advance for any research of this type, finding no similar review on biomarkers in current literature.

### Objective

This systematic review aims to determine the behavior profile of inflammatory biomarkers among patients following elective degenerative spine surgery and their differences compared to those coursing with PSI.

## Methods

### Overview

**Figure 1:**
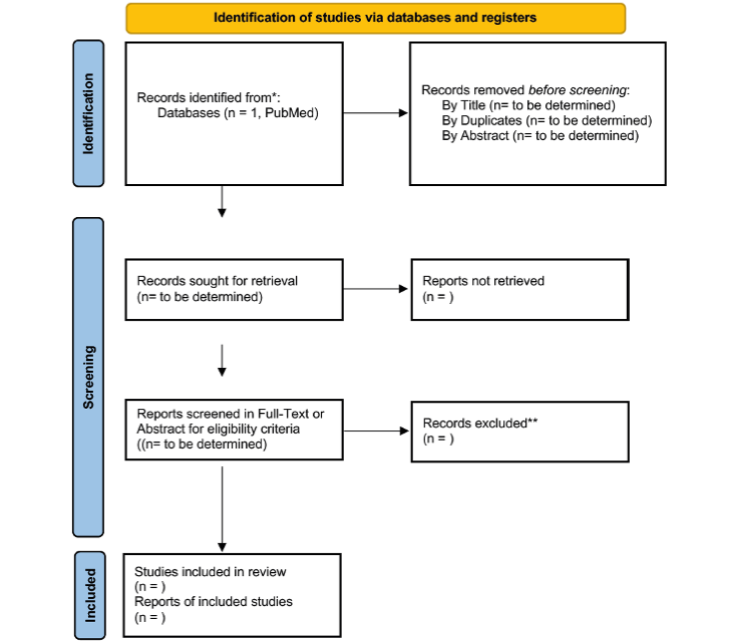
PRISMA (Preferred Reporting Items for Systematic Reviews and Meta-Analyses) flow diagram.

The proposed systematic review will follow the PRISMA (Preferred Reporting Items for Systematic Reviews and Meta-Analyses) statement [23]. This protocol was registered on PROSPERO on January 19, 2022, with registration number CRD42022304645 (February 19, 2022). Figure 1 presents the flow diagram of the methodology.

### Search Strategy

The search strategy will aim to locate all published studies on biomarker evaluation among patients following elective degenerative spine surgery and their differences with those coursing with PSI. An initial search was made on the PubMed database to identify if there was any other review on the topic that would reunite all biomarkers in a single study, finding no one. We will use the PubMed, Embase, and Latindex databases to search among titles and abstracts for the specific word search criteria from 1984 to the present.

Keyword criteria included “Postoperative spinal infection” and a combination of either one of the following: “Biomarker,” “Marker,” “Detection,” “C Reactive Protein,” “CRP,” “Erythrocyte Sedimentation Rate,” “ESR,” “Procalcitonin,” “PCT,” “Retinol Binding Protein,” “Albumin,” “Prealbumin,” “Transferrin,” “Neutrophil/Lymphocyte Count,” “Neutrophil,” “Lymphocyte,” “Dickkopf1,” and “DKK1.” Multimedia Appendix 1 shows the full search strategy for PubMed in detail. We will include papers predominantly in English and other languages where information is readily available.

### Review Question and Keywords

Our review question is as follows: What is the behavior profile of inflammatory biomarkers among patients following elective degenerative spine surgery and their differences from those coursing with a PSI?

Keyword criteria included the following: “Postoperative spinal infection” and a combination of either one of the following: “Biomarker,” “Marker,” “Detection,” “C Reactive Protein,” “CRP,” “Erythrocyte Sedimentation Rate,” “ESR,” “Procalcitonin,” “PCT,” “Retinol Binding Protein,” “Albumin,” “Prealbumin,” “Transferrin,” “Neutrophil/Lymphocyte Count,” “Neutrophil,” “Lymphocyte,” “Dickopf1,” and “DKK1” from January 1, 1984, to June 4, 2022. Multimedia Appendix 1 shows the full search strategy for PubMed in detail. We will include papers predominantly in English and other languages where information is readily available.

### Inclusion Criteria

#### Participants

We will include studies with adult patients (>15 years old), studies related to biomarkers in patients operated on for degenerative spinal diseases, and those developing a PSI, and studies with raw data and means. We will exclude all studies of pediatric patients (<15 years old) and patients operated on for any other etiology such as tumors, trauma, congenital diseases, and so on, and studies where the biomarker parameters could not be obtained within the text, figures, or tables.

#### Concept

The concept is to gather all the information related to the inflammatory biomarker’s behavior profiles among patients following elective degenerative spine surgery and their differences with those coursing from PSI in the perioperative period, the early postoperative period (in hospital) up to 90 days (most agreed time limit to consider an early spinal infection). This information will help us draw a profile curve for biomarkers and each group of patients, aiding in differential diagnosis among them.

#### Context

##### Types of Sources

The review will be limited to Evidence Level IV and above (case series, case-control studies, retrospective, prospective cohorts, randomized and nonrandomized clinical trials, and previous systematic reviews, if any). We will not include editorials, individual case reports, or experimental, nonhuman, or nonclinical studies.

##### Information Sources

We will only conduct this systematic review in the PubMed, Embase, and Latindex databases.

### Study and Source of Evidence Selection

We will collect the full search strategy results in Excel. At this point, the first author (RRJAI) and the second author (FJP) will make the first exclusion process based on titles and duplicates. Later, they will make the second exclusion process based on abstracts. Next, all papers that will require full-text reading will be handled in Mendeley; both authors will read them independently for inclusion criteria; in case of disagreement, we will include papers after a joint decision with the third author (RMWC). Then, the former author will perform the data extraction, and the second author will verify this process; we will also solve any disagreement in a joint decision with the third author. Next, we will record the reasons for exclusion in Excel. Finally, we will report the full results of the search in the final paper and present it using the PRISMA checklist.

### Data Extraction

The first author will perform data extraction manually, and the second author will verify this process. The following information will be extracted from the papers: (1) study title; (2) study author; (3) year; (4) evidence level; (5) research type; (6) diagnosis group (elective postoperative degenerative disease or PSI); (7a) region (cervical, thoracic, lumbosacral, and coccigeal); (7b) type of infection by anatomical or radiological site; (8) surgery type (including instrumentation or not); (9) number of cases; (10) mean age or individual age; (11) individual serum biomarker values from the preoperative state, postoperative day 1 up to 90 days for both groups and also at day of diagnosis (within the 90 postoperative days) for the PSI group including (10a) interleukin-6 (IL-6), (10b) presepsin (PSP), (10c) erythrocyte sedimentation rate (ESR), (10d) leukocyte count, (10e) neutrophil count, (10f) C-reactive protein (CRP), (10g) serum amyloid, (10h) white cell count (WCC), (10i) albumin (Alb), (10j) prealbumin, (10k) procalcitonin (PCT), (10l) retinol-associated protein, and (10m) Dickkopf-1 (DKK1); (11) postoperative days at symptoms or diagnosis; (12) type of organism; (13) day of starting antibiotics; (14) duration of treatment; and (15) any biases (including comorbidities, especially those affecting immunological status).

We will modify the proposed data extraction schema as needed. We will detail any modifications in the full review report. We will solve any disagreements between the reviewers through discussion with the third author.

### Data Analysis and Presentation

We will present the extracted data in a tabular form and as a narrative summary as required. The table will report the following variables: (2) study author; (3) year; (4) evidence level; (5) research type; (6) diagnosis group; (7a) region (cervical, thoracic, lumbosacral, coccigeal); (7b) type of infection by anatomical or radiological site; (8) surgery type (including instrumentation or not); (9) number of cases; (10) mean age or individual age; (11) postoperative days at symptoms or diagnosis; (12) type of organism; (13) day of starting antibiotics; (14) duration of treatment; and (15) any biases (including comorbidities, especially those affecting immunological status).

We will use graphical representations for the following variables to compare the behavior profile of each biomarker concerning time from the preoperative state up to 90 days postoperative among the 2 groups (elective postoperative spine degenerative disease vs PSI): (10a) IL-6, (10b) PSP, (10c) ESR, (10d) leukocyte count, (10e) neutrophil count, (10f) CRP, (10g) serum amyloid, (10h) WCC, (10i) Alb, (10j) prealbumin, (10k) PCT, (10l) retinol-associated protein, and (10m) DKK1.

## Results

No ethical approval will be required, as this review is based on published data and does not involve interaction with human participants. The search for this systematic review commenced in February 2021, and we expect to publish the findings in mid-2023. The plan for dissemination, however, is to publish the review’s findings in a peer-reviewed journal and present findings at high-level international conferences to share knowledge to help establish more timely and accurate diagnoses to benefit patients.

## Discussion

### Overview

PSI is an elusive illness that can be challenging to demonstrate even in the context of advanced laboratory and imaging diagnostic studies [24,25]. Early diagnosis aids in achieving a good prognosis and contains the devastating consequences of spinal infections, reducing morbidity and health care costs [26,27]. Current research is focused on the behavior of serum biomarkers in the early postoperative period [17], where the white blood cell count, CRP, ESR, serum amyloid A, and PCT have been established as the conventional biomarkers [24]. Research for these biomarkers, as well as other new ones, relies primarily on retrospective cohorts on a single-measurement basis [11,22,28-32] with few prospective studies performed up to date [17,24,29], representing a significant limitation in the validity of results. Although several biomarkers exist in current research, they are studied individually [17] for a heterogenous group of illnesses in the spectrum of spinal infection with patients undergoing different surgical procedures [17]. As a result, cutoff values to determine abnormal inflammatory biomarker response in the postoperative period are lacking and vary widely depending on the severity of the illness [17] and the extent of the surgical procedure [33].

To have a point of comparison, we will summarize key findings in each of the biomarkers studied up to date. The acute-phase CRP is the biomarker most studied in the scientific literature related to spinal infections due to its availability and feasibility in clinical practice [34]. Its serum concentrations have served to diagnose spinal infection, monitor response to treatment, and detect relapse [34]. The difficulty in using it to detect PSI is having a trustable cutoff value for the inflammatory response expected from the surgical procedure itself [34], which depends on the extent of the procedure [33] and the severity of the illness [17]. Although no clear cutoff values can be drawn from the conclusion of the research and there exist contrasting evidence, it seems that CRP rises in the second and third postoperative day of noninfected patients, lowering its concentrations thereafter; this rise is dependent on the extent of surgery, but the behavior is similar for both of them [34]. As a result, secondary rises [32] or persistently high CRP serum concentrations beyond this period are suspicious of PSI, specifically after postoperative day 7 [35,36]. Nevertheless, these findings can derive from other noninfectious conditions in the postoperative period or have no obvious explanation [32]. Provided that CPR is most sensitive but not that specific (100% and 91.7%), other biomarkers such as PCT have demonstrated remarkable usefulness in differentiating inflammatory response as a result of surgery from that of spinal infection (100% specificity and 95.2% specificity) [37] provided that PCT does not seem to be affected because of the extent of surgery and rises in the first postoperative day remaining steadily low there forward [37]; unfortunately, it does not differentiate infection in other anatomical regions and there also exists evidence reporting no difference in PCT concentration among patients with and without postoperatory spinal infections [38], adding to the lack of a clear cutoff value to consider infection. The neutrophil-to-lymphocyte ratio is another marker that has constantly demonstrated diagnostic usefulness in the early postoperative period, with reported cutoff values ranging from 3.21 to 3.87 for differentiating postoperatory state from a spinal infection; however, this information is based on retrospective cohorts [25,38]. Finally, many other biomarkers, such as IL-6, retinol-binding protein, prealbumin, and PSP, among others, have been studied with contradictory evidence and no clear practical usefulness [1,11,22,24]. The expression of DKK1 has been recently evaluated in the context of osteomyelitis [39] and infectious processes [40-42] but as far as we know, there exist no reports of spinal infections. We include this marker in the systematic review to confirm that there exist no previous studies in this respect as to establish a starting point for a research line in this direction. We consider DKK1 a valuable marker to be investigated provided that its expression affects bone repair after damage by the Wnt/β-catenin molecular signaling pathway for osteoclast and osteoblast induction, where DKK1 functions as an inhibitory factor (that can be induced by infections), therefore impeding bone renewal [40]. By the relationship DKK1 has with infections and impeding bone repair after damage (such as a surgical insult), we consider it an important factor to be investigated in the context of postoperatory spinal infection.

Therefore, this protocol aims to direct many of these limitations found in previous research. We expect to reunite all the information gathered from previous retrospective and prospective series to provide results based on a large sample. Simultaneously, we expect to obtain the mean serum concentrations for different biomarkers over time from the preoperative period up to the postoperative day 90 by extracting data from different series with different time approaches. By using these 2 strategies, we aim to draw time-dependent behavior profiles for each of the biomarkers to have a robust reference of average values and confidence intervals in the postoperative period and abnormal cutoff values and ranges for PSI. Also, we expect to be able to draw specific cutoff values for the different surgical procedures performed; nevertheless, this objective will be ultimately dependent on finding an appropriate surgical procedure description and the sample size obtained from the included studies. We consider that our findings will help clinicians and surgeons make more evidence-based timely decisions that benefit the patients with early treatment, avoiding long-term complications and increases in health care costs with the ultimate impact on morbidity, disability, and mortality. Additionally, our study will be the first and more robust research in this respect by relating all the biomarkers that have shown utility in the diagnosis of spinal infection on a single systematic review taking into consideration the full perioperative period and the different surgical techniques employed.

Finally, there are also limitations to consider in our research. For example, several studies have an incomplete reporting of results due to data gathering bias, such as a lack of protocol for taking blood samples derived from retrospective research. Also, some retrospective and prospective studies that evaluate biomarkers have directness biases as they are evaluated as a secondary objective that impacts outcomes, prognosis, or are considered among different risk factors. Nevertheless, these limitations are unavoidable based on the available literature.

### Conclusions

In conclusion, this study will provide the biomarkers’ behavior profile for PSI and patients following elective surgery for degenerative spinal diseases from the preoperative period up to 90 days postoperative and provide cutoff values on the day of diagnosis. Our research will hopefully aid in the differential diagnosis of these groups early by summarizing all research done in this respect. It will eventually provide clinicians with a better background to establish their diagnosis based on highly trustable information based on cutoff values from this systematic review. Shortly, we also expect this review to provide a basis for more robust prospective studies and even clinical trials on biomarkers to establish a more accurate and timely diagnosis of the early stages of the disease, which will ultimately impact the patient’s physical and mental health, reducing the burden of disease.

## Appendix

Multimedia Appendix 1 PubMed search strategy.

## Abbreviations

Alb: albumin
CRP: C-reactive protein
DKK1: Dickkopf-1
ESR: erythrocyte sedimentation rate
IL-6: interleukin-6
PCT: procalcitonin
PRISMA: Preferred Reporting Items for Systematic Reviews and Meta-Analyses
PSI: postoperative spinal infection
PSP: presepsin
WCC: white cell count
